# Production, Quality, and Acceptance of *Tempeh* and White Bean *Tempeh* Burgers

**DOI:** 10.3390/foods7090136

**Published:** 2018-08-30

**Authors:** Rayane J. Vital, Priscila Z. Bassinello, Quédma A. Cruz, Rosângela N. Carvalho, Júlia C. M. de Paiva, Aline O. Colombo

**Affiliations:** 1 Faculty of Nutrition, Paulista University—UNIP, Goiânia 74845-090b, GO, Brazil; rayanevitalnutri@gmail.com; 2 Department of Food Science, Embrapa Arroz e Feijão, Santo Antonio de Goiás 75375-000, GO, Brazil; 3 Food Engineering School, Goias Federal University—UFG, Goiânia 74690-900, GO, Brazil; quedma.cruz@gmail.com; 4 Grain and Byproducts Laboratory, Embrapa Arroz e Feijão, Santo Antonio de Goiás 75375-000, GO, Brazil; rosangela.carvalho@embrapa.br; 5 Food Science and Technology School, Paulista University—UNIP, Goiânia 74845-090, GO, Brazil; judepaiva3108@gmail.com; 6 Graduate Program on Food Science and Technology, Goias Federal University—UFG, Goiânia 74690-900, GO, Brazil; colomboaline@yahoo.com.br

**Keywords:** *tempeh*, *Phaseolus vulgaris* L., nutritional value, sensory analysis, *Glycine max* L.

## Abstract

The food industry has been challenged to develop new healthy food products. *Tempeh*, originating in Indonesia and produced by fungal fermentation, would be an alternative healthy food for the Brazilian population. This study was designed to produce white bean (cv BRS Ártico) *tempeh* burger, to determine and compare its nutritional and sensory properties with conventional soybean-based *tempeh* burger. The production and the analyses of proximal composition and microbiological contamination were determined in the *tempeh*, following reference methods. For the sensory analysis, a nine-point hedonic scale test was performed with 82 untrained evaluators, and at the end, a question of purchase intent was answered. The results indicated significant differences in the nutritional value of the *tempehs*, which is justified by the difference in the composition of the raw materials used. The samples did not present a risk of microbiological contamination for consumption. The white bean *tempeh* burgers showed similar appearance and crispy consistency, but received lower scores for flavor, compared to the soybean burgers, probably due to their residual beany flavor. The beany flavor could be minimized by increasing the cooking time of the beans. White bean *tempeh* can be a good alternative for healthy eating, and its manufacture could promote the production of new products made from beans, giving a new focus to the Brazilians’ traditional food. It is still necessary to improve the techniques of production and test new ingredients for the preparation of *tempeh* burgers to obtain higher acceptability.

## 1. Introduction

The food industry has targeted healthy and diversified food for the development of new products in the market all over the world. The fermented food is one example of recent products demanded by a considerable population group whose interest in variability and new foods with functional, nutritional, and tasty attributes has increased lately [1]. *Tempeh* is a traditional Indonesian food, produced by fermentation of soybeans using *Rhizopus* species, having nutritional qualities and metabolic regulation functions [2]. It can also be produced from other substrates, such as beans, corn, rice, lentils, and barley. Brazil, being one of the largest producers, consumers and holders of technologies for bean production, could engage in this promising field by encouraging research on beans [3].

*Phaseolus vulgaris* L. (common beans) is one of the primary sources of protein and one of the essential foods for the Brazilian population. It presents an average protein content of 28% and has all the essential amino acids in its composition; it is rich in lysine, but limiting in sulfur amino acids—methionine and cysteine [4]. Although there is a regional preference for a specific type of beans, those from the *Carioca* group are the 16 most cultivated in Brazil, representing 70% of the national consumption, cultivar Pérola being the most consumed [5]. There is also a growing potential for types of beans with different characteristics of color, shape, and size, attracting gourmet gastronomy for different culinary preparations and in the food industry, not only as the traditional cooked beans [6]. Fermentation of leguminous seeds as beans has several advantages, since it reduces non-nutritional factors, improves nutrient digestibility, reduces allergenicity, activates antioxidant activity, and the concentration of phenolic compounds can be increased during the fermentation process, in addition to being associated with the reduction of chronic diseases risk. Therefore, there is a growing interest in promoting the production of fermented leguminous seeds [7].

The 68th United Nations General Assembly declared 2016 the International Year of Pulses to raise public awareness of the nutritional benefits, sustainable production, food security, nutrition, creating an opportunity to encourage better utilization of plant proteins, crop rotation and the trade of “pulses” [8]. In this context, there are countries in Africa and Asia, such as Indonesia and India, where *tempeh*, due to its nutritional and sensory properties and versatility in the preparation in pure form or as an ingredient in a number of other food preparations (hamburgers—“green meat”, vegetarian products, lyophilized or roasted *tempeh* flour for biscuits), has been stimulated in public policies, as an alternative in the multimixtures to fight the malnutrition of deprived populations, especially in mothers and children undergoing weaning [9].

The objective of this work was to develop *tempeh* and *tempeh* burgers from white beans (cv BRS Arctic) without tegument by solid fermentation and to compare their nutritional and sensorial characteristics with conventional soybean *tempeh* burgers.

## 2. Materials and Methods

### 2.1. Acquisition of the Material

Beans of cultivars BRS Arctic and conventional soybeans BRS 284 used in this study were from the 2015 harvest season at Fazenda Capivara, Embrapa Arroz e Feijão/GO/Brazil. The *Rhizopus oligosporus* strain was purchased from the Tropical Cultures Collection of André Tosello Research and Technology Foundation, Campinas/SP. Samples of soybean tempehs, commercial products of the same lot, were purchased from Totale Tempeh manufacturer at Rezende/RJ/Brazil and used as reference.

### 2.2. Raw Material Preparation

Dry beans and soybeans were homogenized, sorted manually, and only the whole and healthy ones were selected, placed in polyethylene bags, and stored in a cold room until use.

### 2.3. Rhizopus oligosporus Multiplication

*Rhizopus oligosporus* strains were transferred to Petri dishes with PDA (Potato Dextrose Agar) medium for increased spore production. After mycelial growth and spore formation, the surface of each plate was scraped with a platinum handle and the mycelium transferred to an Erlenmeyer flask containing 100 mL of sterile distilled water and counting performed in the Neubauer chamber, as reported by Miyaoka [10]. To determine the ideal culture medium for inoculum production, the *Rhizopus* strain was seeded in Petri dishes containing the following media: Potato Agar Dextrose (PDA), PDA + rice flour, PDA + bean flour, PDA + 50% rice flour + 50% bean flour. The autoclaved culture media were inoculated in a laminar flow chamber and incubated in an oven at 30 °C for 48 h. The medium presenting the best fungus development was chosen for inoculum preparation.

### 2.4. Spore Counting

Spore counts were performed using the Neubauer chamber (Global Trade, Double Improved—BSN 020; Hatfield, PA) where 0.2 mL of the suspension was homogenized and transferred to a test tube and 0.6 ml of 0.2% trypan blue dye was added. A 0.5 mL aliquot was then placed on a cover plate for spore counting under the microscope. For calculation of number of cells/mL, Equation (1) was used:

Number of cells/mL = (total number of cells × dilution factor × 10,000)/number of quadrants counted
(1)

### 2.5. Inoculum Preparation

For the production of the flour inoculum, 100 g of type 1 rice were grounded and sieved on a BERTEL^®^ vibrating sieve (Caieiras, SP, Brazil) using 200 mesh sieves. The flour was placed in glass containers with metal lid, sterilized, and cooled at room temperature. Twenty milliliters of the inoculum was inoculated into each vessel and incubated in an oven at 30 °C for 5 days. After this period, the containers were refrigerated and used for up to 30 days.

### 2.6. Tempeh Production

**Figure 1:**
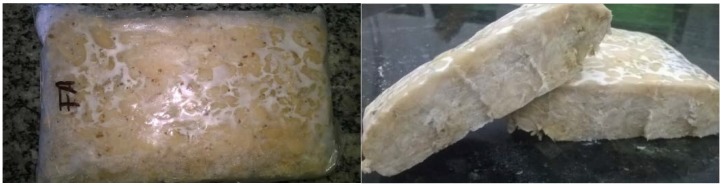
*Tempeh* of white beans after mycelium total development.

*Tempehs* were prepared following the methodology used by Starzynska-Janiszewska et al. [11]. Two hundred grams of beans were cleaned in running water, submerged in 1000 mL of sterilized water at room temperature for hydration/maceration with the addition of 20 mL of commercial alcohol vinegar containing acetic acid (5%) for 20 h. For the removal of surface water, beans were dried on paper towels at room temperature and the tegument removed manually. Heat treatment was performed by conditioning the beans in beakers capped with laminated paper, autoclaving them for 15 min at 121 °C, and then draining them and cooling at room temperature. After those procedures, beans were placed in polyethylene bags and inoculated with 20 g of the inoculum previously produced with rice flour and strain of *Rhizopus oligosporus*. The bags were sealed and small holes were made with a fork to allow the contact of the fungus with oxygen. Finally, beans were incubated in an oven at 30 °C and visually monitored to follow up on the development of the mycelium (about 30 h) (Figure 1).

### 2.7. Analytical Determinations

White bean and soybean *tempehs* samples were dehydrated by lyophilization in a LIOTOP^®^ L101 lyophilizer equipment (São Carlos, SP, Brazil) for 48 h until all material was completely dehydrated. The nutritional characterization was performed by official methods according to the Association of Official Analytical Chemists (AOAC) 2010 [12]. The moisture content was determined by oven-drying at 105 °C until constant weight; the ash content was evaluated by the gravimetric method of incineration in a muffle oven at 500 °C; the lipid content was determined by continuous extraction in a Soxhlet apparatus using ethyl ether as solvent; the total nitrogen content was obtained by the micro-Kjeldahl method using the factor 6.25 for conversion into protein; the total dietary fiber was analyzed by the gravimetric-enzymatic method established by AOAC 2005 [13] and adapted by Embrapa Agroindústria de Alimentos [14], and the carbohydrate content was calculated by difference, as provided by the Resolution of the Collegiate Board of Directors (RDC) No. 360, December 2, 200,315 by Equation (2):

% carbohydrate = 100 − (% ash + % protein + % lipid + %fiber)
(2)

The energy value of the product was estimated using Atwater conversion factors of 4 kcal/g for protein and carbohydrate and 9 kcal/g for lipid [15].

### 2.8. Microbiological Analysis

The presence of the following microorganisms in the ready-made *tempehs* was analyzed as follows: Coliforms, Staphylococcus positive coagulase and *Salmonella* sp. at 45 °C. The microbiological protocol followed the methodology established by the Compendium of Methods for the Microbiological Examination of Foods [16]. For all analyzed microorganisms, a control test (incubation of the culture medium in a petri dish, without inoculation) was performed to verify the innocuity.

### 2.9. Hamburgers Preparation

For the sensorial analysis, hamburgers were chosen as an alternative for using *tempeh*, because that food is easy to handle and popular among the surveyed public. The same additional ingredients and amounts (Table 1) were used in the formulation of both *tempehs*. They were homogenized in a Mondial^®^ culinary multiprocessor (Brasília, DF, Brazil), manually molded, and grilled in a nonstick frying pan with olive oil until golden brown on both sides. Each 100 g of *tempeh* yielded an average of 11 small burgers.

### 2.10. Tempeh Burgers Sensory Evaluation

*Tempeh* burgers were evaluated by 82 nontrained participants for their appearance, aroma, flavor, and overall impression, using a nine-point hedonic scale (9—I liked very much, 8—I liked it a lot, 7—I liked it moderately, 6—I liked it slightly, 5—I did not like it or disliked, 4—I disliked it slightly, 3—I Disliked it moderately, 2—I did not like it much and 1—I did not like it very much). The tasters were also asked about their purchase intent (I would definitely buy this product, I would probably buy this product, I’m not sure if I would buy this product, I probably would not buy this product, I certainly would not buy this product). The participants were students from the Department of Pharmacy of the Metropolitan College of Anápolis—GO, employees of the Embrapa Rice and Beans Research Center and students from the Federal University of Goiás. They were over 18-year-old nonsmokers, healthy nonpregnant women and men, randomly selected. The participants filled out a Term of free consent and the Consolidated view of the research ethics committee of the Federal University of Goiás, and before receiving the samples were instructed how they should conduct the test. The sensorial analysis was carried out in the Food Technology Laboratory at the Metropolitan College of Anápolis, GO and in the Experimental Kitchen Laboratory at Embrapa Rice and Beans Research Center, Santo Antônio de Goiás, GO. The laboratories have sensory booths for analysis, as well as adequate lighting and kitchen support.

Two samples were made available, the first one being of white bean *tempeh* and the second of soybean *tempeh*. A questionnaire was distributed along with a glass of water and samples served in disposable dishes. The project was submitted to the evaluation and approval of the Research Ethics Committee of the Federal University of Goiás under protocol No. 60631116.6.0000.5083.

### 2.11. Statistical Analysis

Results were analyzed by ANOVA and F test using SISVAR^®^ software, followed by independent 2-group *t*-test using software-R (*p* < 0.05) for comparison of the mean values obtained in the different treatments. For sensory evaluation of mean descriptive values, Tukey test (*p* < 0.05) was applied instead.

## 3. Results and Discussion

### 3.1. Microbiological Analysis

Hygienic-sanitary care during the food manufacturing process is a preventive measure of microbiological contamination and has been a concern of the sanitary inspection agencies. All food must have its quality proven by tests that justify its innocuity [17]. After the production of the *tempehs*, the microbial investigation of the samples was carried out and the results found are expressed in Table 2.

From the data obtained, it can be observed that results for the three microorganisms studied were within the standards established by RDC No. 12, which includes values for Colony-Forming Units (CFU) in fermented foods, thus proving that there was no significant growth of typical colonies of bacteria that could affect the microbiological quality of the product, making *tempeh* safe for consumption. Autoclaving and acidification of the medium, steps used in this study, are useful techniques to control *tempeh* contaminating agents, but it is worth mentioning that good manufacturing practices also contribute to the absence of pathogenic bacteria, as well as storage conditions.

### 3.2. Nutritional Characterization

The nutritional value of the *tempehs* is naturally different due to the composition of the raw material (Table 3).

It is important to emphasize the significant difference observed in the lipid analyses, where the soybean *tempeh* presented 24.88 g/100 g, while that of white bean 1.29 g/100 g, and this difference impacts and explains the significantly different caloric values of both *tempehs*. The white bean *tempeh* sample showed fewer calories because of the lower lipid and intermediate protein contents compared to the soybean *tempeh*. According to Astuti et al. [18], the protein content of soybean *tempehs* and soybeans are practically the same. Due to the action of the protease enzyme produced by the fungus during fermentation, the soluble protein content increases markedly. The soluble nitrogen content in unfermented soybeans is 3.5 mg/g, compared to 8.7 mg/g in *tempehs.* Beans are an excellent food, providing essential nutrients, such as iron and calcium, carbohydrates, and fibers. They are the primary source of protein for the Brazilian low-income population, but the digestibility of these proteins is relatively low. According to Mesquita et al. (2007), the protein value of the white bean *tempeh* does not increase significantly after fermentation, and the value here was 23.34 g/100 g [19]. However, it is reported in the literature that *Rhizopus* uses amino acids as a source of nitrogen for its growth. This might suggest that the total amino acid content decreases, but free amino acids increase, making white bean *tempeh* protein probably more digestible than cooked beans.

### 3.3. Total Food Fiber

Regarding total fiber content, white bean and soybean *tempehs* showed no significant difference, except for the insoluble fiber fraction (Table 4).

According to FAO/WHO, an adult individual needs about 25 grams of fiber per day, which makes *tempeh* products interesting for fiber supply, and they may also act in the control of intestinal transit and the treatment of comorbidities such as obesity.

## 4. Sensory Analysis

**Figure 2:**
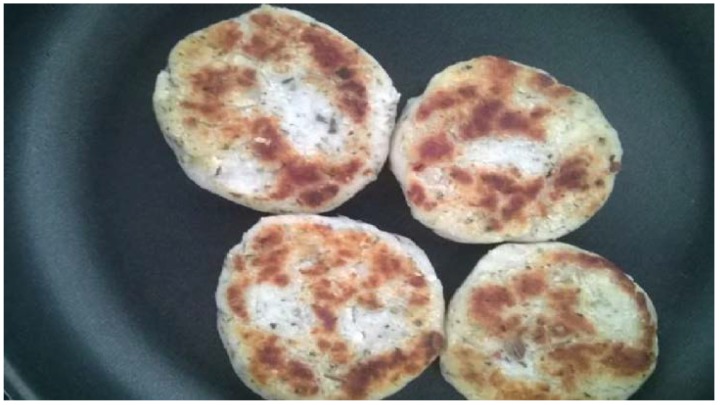
White bean *tempeh* burger.

Hamburgers of both *tempehs* presented a firm and consistent shape, similar appearance, pleasant odor, a brownish crust and a certain degree of crunchiness (Figure 2).

For appearance, 68.29% of the participants liked the white bean *tempeh* burger, and 23.17% liked it very much. Not very dissenting results were obtained for the soy burger, where 74.39% of the participants reported liking the appearance of the product, and 28% liked it very much. Among these, 16 evaluators disliked the white bean *tempeh* hamburger and eight, the soy one. The aesthetic parameter is one of the central questions taken into account by the consumer when assessing the safety of food, which incorporates a different concept of food safety, taking into account technical concepts such as odor, nutritional value, and appearance. The appearance of the *tempeh* burgers was considered to be nice, resembling chicken burgers.

For flavor, 47.56% of the participants reported liking the white bean *tempeh* burger, with only 6% of the individuals showing a great liking for the product; a very different result for the soy *tempeh* hamburger, where 68.29% liked the product at different intensities, and 24.39% showed great liking. Of the total participants, 34.15% disliked the white bean *tempeh* burger and 20.73% the soy one. This can be explained by the fact that the aroma of the bean is not part of the olfactory memory linked to the aroma of hamburgers. When one imagines or visualizes a food, the aroma, and the flavor are automatically sought in the subconscious, and when one tastes it one expects an aroma that is already similar to the known [20]. The storage conditions of the beans and even the ready-made *tempeh* may have resulted in the loss of quality causing the off flavor due to the oxidation of the beans’ unsaturated fatty acids. The unpleasant taste and odor in the soy products are attributed to the action of lipoxygenase enzymes, which form hydroperoxides from polyunsaturated fatty acids [21].

For flavor evaluation, 39.02% of the participants said they liked the white bean *tempeh* burger, and 53% liked the soy one. Of the total participants, 35 of them disliked the white bean *tempeh* burger, and only 17 disliked the soybean. The same considerations regarding aroma are applied to flavor since this attribute is a mixture of olfactory, gustatory, and tactile sensations. There were reports of the presence of a residual flavor in both burgers. The remaining taste in the mouth sometime after the food has been swallowed seems to be more noticeable in the white bean burger. The autoxidation of the fatty acids present in the soybean generates several classes of volatile compounds, which contribute to the residual taste of ‘green grass’. One hypothesis for the low acceptance of the soy and white bean *tempeh* burgers would be their manufacturing process, which had a shorter cooking time compared to soybeans and home-cooked beans. The overall impression (Table 5) depicts a collection of prejudged elements that define the appraiser’s appreciation of the product as a whole. In this case, the soy *tempeh* burger obtained higher scores than the white bean *tempeh* one, presenting a higher performance in all attributes.

**Figure 3:**
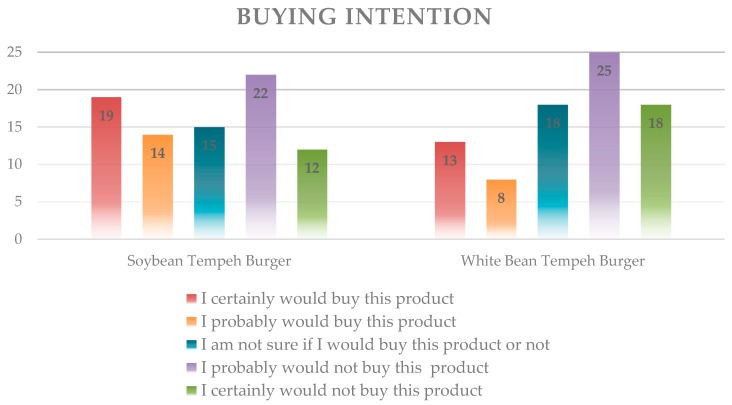
Purchase intention for the *tempeh* burger.

In general, the soybean burger had a higher average score for all attributes, but there was no significant difference in appearance, demonstrating that both burgers were similar. Figure 3 shows that the buying intention for the soy *tempeh* burger was higher than that for the white bean *tempeh* burger, which is consistent with the attributes previously analyzed.

Burgers can be one of several options for the use of *tempeh.* The low purchase intent may be associated with the fact that the consumers connect the hamburger with characteristics of succulence, meat flavor, frying smell, and darker color. Shurtleff et al. [22], in the mid-1980s, mentioned that soybean burgers were used in campaigns in Europe to promote the consumption of soy-based products as a beef substitute and as a healthy food. When faced with the bean samples, the expectation created for the consumption of this food is dissolved by the difference from traditional hamburgers.

## 5. Conclusions

White bean *tempeh* is an innovative food; it has good nutritional value, with a considerable amount of protein; may be an alternative and eventually an option for meat, and can be consumed by vegetarians and sympathizers. It also has a high content of carbohydrates, calories, and a good source of fibers, being an excellent product for energy intake, and if inserted in a balanced diet, it may act as intestinal regulator. The soybean *tempeh* burger showed higher scores in all attributes evaluated in the sensory analysis, demonstrating the need for further research to either improve *tempeh* production techniques and to use other ingredients for the preparation of hamburgers or other *tempeh* products to provide greater acceptance of this new product. White bean *tempeh* could be a good alternative for healthy eating, but its recommendation should be based on scientific studies which demonstrate its beneficial effects. Continuous scientific research is necessary to identify beneficial components, their mechanisms of action, function, nutritional aspects. The production of legume-based *tempeh* can promote the creation of new products made from common beans, giving an alternative to the traditional Brazilian food.

Finally, we believe that this study has some potential social and economic impacts such as the contribution to the advancement of scientific knowledge regarding the pioneering process of manufacturing *tempeh* in Brazil; it gives nutritionists the opportunity to explore the versatility of common beans in gastronomy; improves the bean production chain as well as the small farmer’s techniques, and gives them the opportunity to explore different common bean cultivars.

## Figures and Tables

**Table 1:** Additional ingredients for 100 g *tempeh* burger preparation.

Ingredients	Amount
Salt	2 g
Pepper	2 g
Dehydrated onion	3 g
Dehydrated garlic	3 g
Dehydrated parsley	4 g
Dehydrated green onions	4 g
Olive oil	50 mL

**Table 2:** Evaluation of the microbiological contamination of *tempehs*.

Identification	Coliforms 45 °C/g	Staph. Positive Coag./g	*Salmonella* sp./25 g
White bean *Tempeh*	≤10 CFU	≤10 CFU	Absence in 25 g
Soybean *Tempeh*	≤10 CFU	≤10 CFU	Absence in 25 g
Microbiological Reference Limits *	10^2^ CFU/g	10^2^ CFU/g	Absence in 25 g

* Source: Adapted from Resolution of the Collegiate Board of Directors (RDC) No. 12, 2 January 2001, for fermented foods.

**Table 3:** Proximal composition * and calories content of white bean and soybean *tempehs*.

Identification	Moisture (%)	Ash (g/100 g)	Lipid (g/100 g)	Protein (g/100 g)	CHO (g/100 g)	Kcal/100 g
White bean *tempeh*	3.94 ± 0.10 ^a^	2.40 ± 0.08 ^a^	1.29 ± 0.04 ^b^	23.34 ± 0.21 ^b^	55.45 ^a^	326.77 ^b^
Soybean *tempeh*	2.57 ± 0.12 ^b^	2.03 ± 0.01 ^b^	24.88 ± 0.30 ^a^	43.74 ± 0.28 ^a^	10.39 ^b^	440.44 ^a^

* Means from three determinations ± standard deviation followed by the same lowercase letter in the same column do not differ by the *t*-test at 5% significance (*p* < 0.05).

**Table 4:** Dietary fiber fractions in white bean and soybean *tempehs* *.

Identification	Soluble Dietary Fiber (g/100 g)	Insoluble Dietary Fiber (g/100 g)	Total Dietary Fiber (g/100 g)
White bean *Tempeh*	4.11 ^a^	13.40 ^b^	17.52 ^a^
Soybean *Tempeh*	2.18 ^a^	16.80 ^a^	18.96 ^a^

* Same lowercase letters in the same column do not differ by the *t*-test at 5% significance (*p* < 0.05).

**Table 5:** Overall scores of the sensorial analysis of white bean and soybean *tempeh* burgers *.

Identification	Appearance	Aroma	Flavor	Overall Impression
White bean *tempeh*	6.22 ^a^	4.00 ^b^	3.55 ^b^	5.10 ^b^
Soybean *tempeh*	6.93 ^a^	6.54 ^a^	6.35 ^a^	6.40 ^a^

* Same lowercase letters in the same column do not differ by the Tukey test at 5% significance (*p* < 0.05).
